# Covalently Immobilized Mitomycin C on Polypropylene Sutures Creates a Non-Releasing Bioactive Interface That Modulates Vascular Smooth Muscle Cell Fate and Prevents Intimal Hyperplasia

**DOI:** 10.3390/ijms27031328

**Published:** 2026-01-29

**Authors:** Tzu-Yen Huang, Wei-Chieh Chiu, Ko-Shao Chen, Ya-Jyun Liang, Pin-Yuan Chen, Yao-Chang Wang, Feng-Huei Lin

**Affiliations:** 1 Institute of Biomedical Engineering, College of Medicine and College of Engineering, National Taiwan University, No. 1, Sec. 4, Roosevelt Rd, Taipei City 10617, Taiwan; d07528011@gmail.com (T.-Y.H.);; 2 Department of Thoracic and Cardiovascular Surgery, Chang Gung Memorial Hospital at Keelung, 222 Mai-Chin Road, Keelung 204201, Taiwan; 3 Department of Materials Engineering, Tatung University, No. 40, Sec. 3, Zhongshan N. Rd., Taipei City 10452, Taiwan; 4 Department of Neurosurgery, Chang Gung Memorial Hospital at Keelung, 222 Mai-Chin Road, Keelung 204201, Taiwan; 5 School of Medicine, Chang Gung University, No. 259, Wenhua 1st Rd., Guishan Dist., Taoyuan City 33302, Taiwan; 6 National Health Research Institutes, 35, Keyan Road, Zhunan Town, Miaoli County 35053, Taiwan

**Keywords:** Mitomycin C, covalent surface grafting, non-releasing biointerface, contact-dependent bioactivity, vascular anastomosis, intimal hyperplasia

## Abstract

Intimal hyperplasia (IH) at vascular anastomosis sites arises from endothelial injury, thrombin activation, and the subsequent proliferation and phenotypic modulation of vascular smooth muscle cells (VSMCs). Existing clinically used systemic pharmacologic regimens (e.g., antiplatelet/anticoagulant therapy) and reported local material-based strategies in the literature (e.g., drug-eluting sutures, hydrogels, or coatings) largely rely on drug release, which can result in burst kinetics, finite duration, and off-target/systemic exposure. We developed a covalently immobilized, non-releasing biointerface in which mitomycin C (MMC) is stably anchored onto polypropylene sutures via low-pressure, non-thermal acetic-acid plasma (AAP) activation. AAP functionalization introduced reactive oxygen-containing groups on polypropylene, enabling amide-bond immobilization of MMC while preserving suture mechanics. Anchored MMC exhibited potent contact-mediated regulation of VSMC fate, reducing metabolic activity to 81% of control, suppressing G_2_/M progression, and inducing a dominant sub-G1 apoptotic population (66.3%), consistent with MMC-induced DNA crosslinking, p21 upregulation, and cyclin B1–CDK1 inhibition. In vivo, in a rat infrarenal aortic anastomosis model (male Wistar rats, 10–12 weeks, 300–350 g), MMC-anchored sutures markedly reduced arterial wall thickening and α-SMA and PCNA accumulation at 4 and 12 weeks, without overt evidence of systemic toxicity. Notably, no measurable MMC release was detected under the tested conditions, supporting that the observed bioactivity is consistent with an interface-confined mechanism rather than bulk diffusion. This work establishes a non-releasing suture-based platform that delivers sustained molecular regulation of vascular healing through interface-confined control of VSMC behavior. Covalent drug anchoring transforms a clinically used suture into an active therapeutic interface, providing a promising strategy to prevent pathological vascular remodeling and anastomotic IH.

## 1. Introduction

Vascular anastomoses are fundamental to cardiovascular, peripheral, and microvascular surgery and directly determine graft patency, hemodialysis access durability, and tissue survival [1,2,3,4,5,6,7,8]. Despite technical advances, postoperative intimal hyperplasia (IH) remains a significant cause of late stenosis and graft failure [9,10,11]. IH is initiated by endothelial disruption and thrombin activation, followed by platelet adhesion, inflammation, and the release of growth factors that drive VSMC proliferation, migration, extracellular matrix deposition, and phenotypic switching from contractile to synthetic states [12,13,14,15,16]. Upregulation of proliferating cell nuclear antigen (PCNA) and α-smooth muscle actin (α-SMA) reflects this pathological transition.

Systemic antiplatelet or anticoagulant therapy is clinically used to mitigate thrombosis but offers limited spatial control over localized molecular events at the anastomotic interface and increases bleeding risk [17,18,19]. Accordingly, a range of local, material-based approaches has been explored, including drug-eluting sutures and luminal graft coatings that release antiproliferative agents to suppress neointimal formation [20,21,22,23,24,25]. Representative preclinical studies of drug-eluting sutures have reported reduced neointimal hyperplasia in rodent vascular models, supporting the feasibility of local pharmacologic modulation at the suture–tissue interface [20,23]. However, most local delivery platforms remain diffusion-driven and are therefore constrained by burst kinetics, finite drug reservoirs, and uncertain long-term dose–time control [20,24]. These limitations are particularly relevant for sutures, which experience cyclic deformation and friction that can compromise coating integrity and durability under physiological conditions [16,24]. In addition, poorly localized or prolonged elution may raise concerns regarding off-target exposure and potential interference with endothelial healing, which is critical for long-term patency [26,27]. In contrast, non-eluting, surface-immobilized biointerfaces offer an alternative paradigm in which bioactivity is confined to the cell–material interface and mediated by direct contact rather than bulk diffusion [28,29,30,31,32]. To address these gaps, we pursued a contact-dependent, non-releasing strategy enabled by acetic-acid plasma (AAP) surface functionalization of clinically used polypropylene sutures [33,34].

Specifically, we developed a non-releasing molecular biointerface in which mitomycin C (MMC) is covalently immobilized onto polypropylene sutures via AAP functionalization. AAP activation introduces oxygen-containing groups that permit stable MMC anchoring without compromising mechanical integrity [33,34]. Rather than relying on drug diffusion, this approach leverages contact-dependent regulation of cellular behavior to enable sustained control of VSMC proliferation at the anastomotic interface.

MMC is a DNA crosslinking agent that induces G_2_/M arrest and apoptosis by inhibiting cyclin B1–CDK1 signaling and activating p21 pathways [35,36,37,38,39,40]. We hypothesized that covalently immobilized MMC would modulate VSMC fate through direct interface interaction while maintaining cytocompatibility per International Organization for Standardization (ISO) 10993-5 (ISO 10993-5:2009; Biological evaluation of medical devices—Part 5: Tests for in vitro cytotoxicity. International Organization for Standardization: Geneva, Switzerland, 2009) and avoiding systemic exposure.

Although MMC is covalently immobilized in this system, its bioactivity is preserved through surface-tethered, contact-dependent molecular interactions [28,29]. Previous studies have demonstrated that surface-bound cytotoxic agents can initiate interface-mediated signal transduction, whereby intimate cell–material contact permits localized membrane perturbation, intracellular uptake of reactive intermediates, or activation of stress-response pathways leading to DNA damage signaling and cell cycle arrest [30,31,32]. In this context, the immobilized MMC functions as a molecularly active biointerface, enabling spatially confined regulation of vascular smooth muscle cell fate without systemic diffusion, thereby establishing a platform for interface-mediated molecular crosstalk between biomaterials and cellular signaling machinery.

Because the MMC layer is covalently tethered, its bioactivity is expected to be interface-confined and therefore dependent on intimate cell–material contact rather than diffusion-driven exposure. Prior studies support the notion that surface-immobilized bioactive moieties can elicit contact-mediated responses through interfacial membrane interactions, and MMC is also known to undergo redox cycling, capable of generating reactive oxygen species (ROS) under bioreductive conditions. These considerations motivated our contact-dependent design and are further discussed in detail in Section 3.1.

Here, we demonstrate that MMC-anchored sutures constitute a stable, non-releasing molecular interface that regulates VSMC behavior and suppresses IH in vivo. This strategy converts an inert, clinically used suture into a long-acting therapeutic system, offering a scalable platform with translational potential for vascular surgery.

## 2. Results

### 2.1. Plasma Functionalization Enables Stable MMC Immobilization

**Figure 1:**
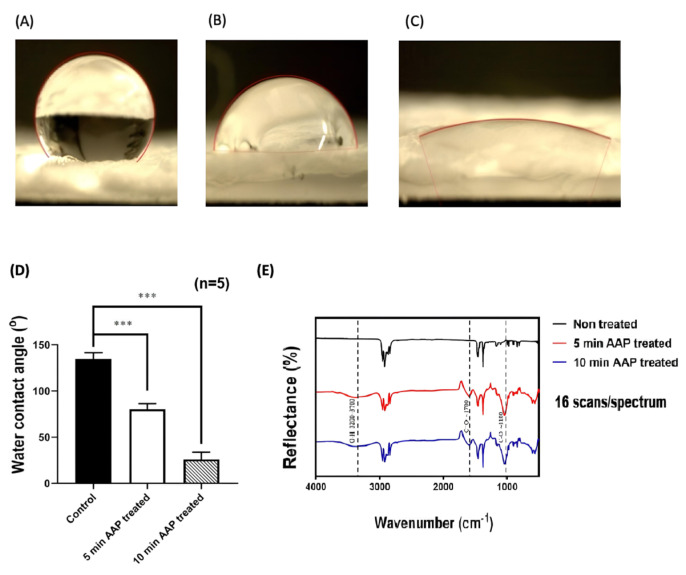
Physicochemical characterization of polypropylene (PP) surfaces following acetic-acid plasma (AAP) treatment. (**A**) Untreated PP surface; PP surface treated with AAP for (**B**) 5 min and (**C**) 10 min (representative water contact angle images showing increased hydrophilicity with longer plasma exposure). (**D**) Quantitative comparison of water contact angle (mean ± standard deviation (SD), *n* = 5) following plasma exposure. (**E**) attenuated total reflection Fourier-transform infrared (ATR-FTIR) spectra (500–4000 cm^−1^; 4 cm^−1^ resolution; 16 scans per spectrum) showing the introduction of oxygen-containing functional groups (O–H, C=O, and C–O) after AAP treatment. The non-treated PP spectrum shows characteristic PP C–H stretching (~2850–2950 cm^−1^) and CH_3_ bending (~1350–1450 cm^−1^) bands. *** indicates *p* < 0.001 (one-way ANOVA with post hoc test).

**Figure 2:**
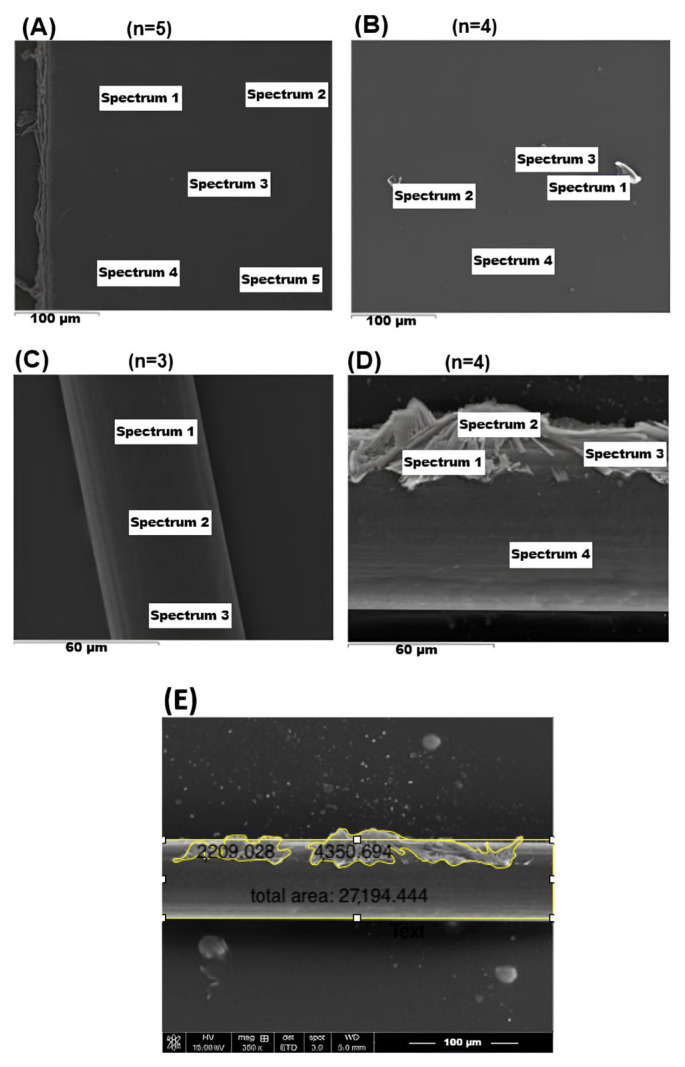
Surface morphology and elemental composition of MMC-grafted polypropylene membranes and sutures. (**A**,**C**) SEM images of plasma-treated PP membrane and suture showing smooth surfaces. (**B**,**D**) Corresponding MMC-grafted surfaces exhibiting particulate and flaky features consistent with immobilized MMC. (**E**) Quantitative surface coverage analysis of MMC aggregates. The labeled regions (“Spectrum 1–5”, as applicable) denote the EDS acquisition sites. Elemental analysis confirms nitrogen incorporation derived from MMC, absent in untreated polypropylene.

The reduction in contact angle from 134.6° to 26.0° confirmed substantial hydrophilization after plasma treatment. Fourier-transform infrared (FTIR) spectra showed characteristic oxygen-containing peaks [33,34] (Figure 1). In the spectrum of the non-treated polypropylene, the characteristic hydrocarbon fingerprint of PP was observed, including C–H stretching bands at ~2850–2950 cm^−1^ and CH_3_ bending bands at ~1350 and ~1450 cm^−1^, indicating preservation of the PP backbone. After AAP treatment, additional absorption features appeared at ~3200–3700 cm^−1^ (O–H stretching), ~1700 cm^−1^ (C=O stretching), and ~1100 cm^−1^ (C–O stretching), consistent with the introduction of oxygen-containing functional groups on the surface. Scanning electron microscopy (SEM) revealed particulate features on treated surfaces, and energy-dispersive X-ray spectroscopy (EDS) detected nitrogen incorporation absent from native polypropylene (Figure 2, Table 1).

### 2.2. Mechanical Performance Remains Intact

**Figure 3:**
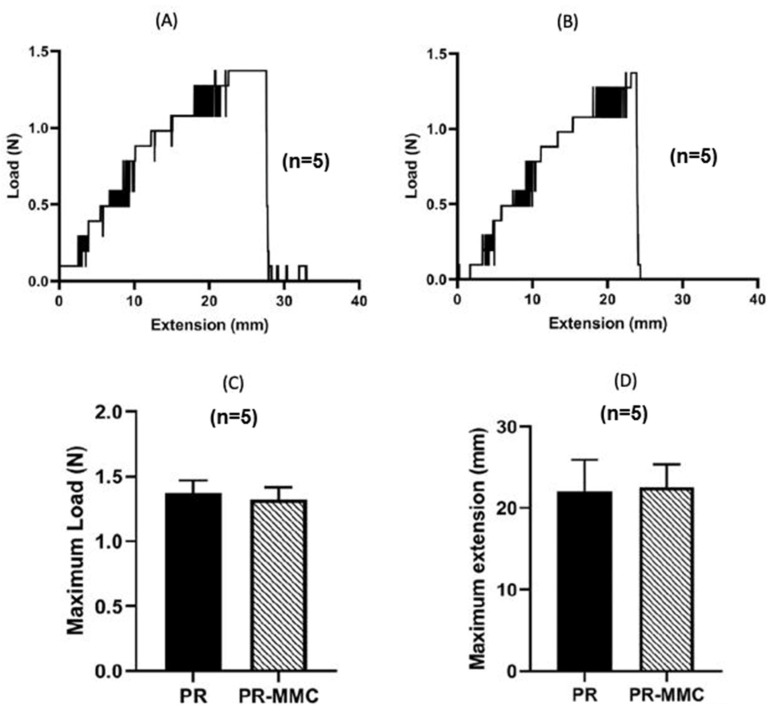
Mechanical integrity of polypropylene sutures following plasma treatment and MMC immobilization. (**A**,**B**) Representative load–extension curves of unmodified and MMC-grafted sutures. (**C**,**D**) Quantitative comparison of maximum load and elongation at break (mean ± SD, *n* = 5), demonstrating preservation of tensile strength and handling properties.

The maximum load and elongation of PR-MMC sutures did not differ significantly from those of unmodified controls (*n* = 5), indicating that handling characteristics were preserved (Figure 3).

### 2.3. Mitomycin C Elution Test by Ultraviolet–Visible (UV–Vis) Spectroscopy

Using the established MMC calibration at 365 nm (A365 = 3.5249·C + 0.0367; R^2^ = 0.9999), the daily collected supernatants from PR-MMC sutures exhibited absorbance values indistinguishable from the blank/background throughout the 14-day monitoring period. Accordingly, MMC signals were not observable above the background under the tested conditions, supporting an interface-confined (non-eluting) bioactive layer rather than bulk diffusion-driven release.

### 2.4. MMC-Anchored Biointerface Triggers Apoptosis and Suppresses the Proliferative VSMC Phenotype

#### 2.4.1. Cytocompatibility (ISO 10993-5)

**Figure 4:**
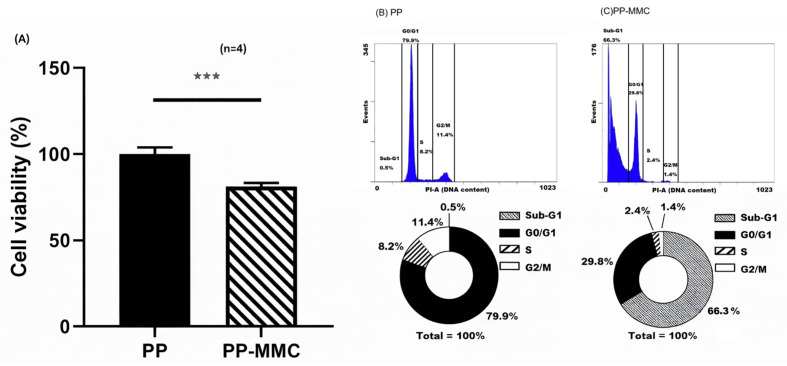
Interface-mediated regulation of vascular smooth muscle cell fate by MMC-grafted surfaces. (**A**) Water-soluble tetrazolium salt-1 (WST-1) assay showing reduced, but ISO 10993-5-compliant, cell viability following co-culture with MMC-grafted membranes. (**B**,**C**) Representative PI-based DNA-content histograms and corresponding gated cell cycle/apoptosis fractions. The *x*-axis denotes propidium iodide (PI)-A (DNA content), and the *y*-axis denotes Events. Vertical gates define the Sub-G1, G0/G1, S, and G2/M regions; percentages indicate the fraction of events within each gate. (**B**) VSMCs cultured with untreated PP show a minimal Sub-G1 fraction. (**C**) MMC-grafted surfaces markedly increase the Sub-G1 fraction and reduce proliferative phases, consistent with contact-dependent induction of apoptosis/cell cycle suppression. Percentages shown in the pie charts are rounded to one decimal place; therefore, the totals may not equal 100%. *** indicates *p* < 0.001 (Student’s *t*-test).

MMC-grafted membranes reduced VSMC metabolic activity to 81.3% (*p* < 0.001) while remaining above the 70% cytocompatibility threshold (Figure 4A).

#### 2.4.2. Cell Cycle Arrest and Apoptosis

Flow cytometry revealed profound shifts in VSMC fate. Control membranes exhibited only 0.5% sub-G1 apoptotic cells, whereas MMC-grafted surfaces induced 66.3% sub-G1 accumulation, consistent with DNA crosslinking–mediated apoptosis [35,36,37,38,39,40]. G_2_/M suppression further indicated inhibition of the cyclin B1–CDK1 pathway (Figure 4B,C).

### 2.5. MMC-Grafted Sutures Attenuate Intimal Hyperplasia Through Suppression of VSMC Proliferation and Phenotypic Switching In Vivo

#### 2.5.1. Systemic Safety

Hematology and blood chemistry values were within or close to normal physiological ranges, with no consistent treatment-related trends across groups or time points (Table 2).

#### 2.5.2. Histology

**Figure 5:**
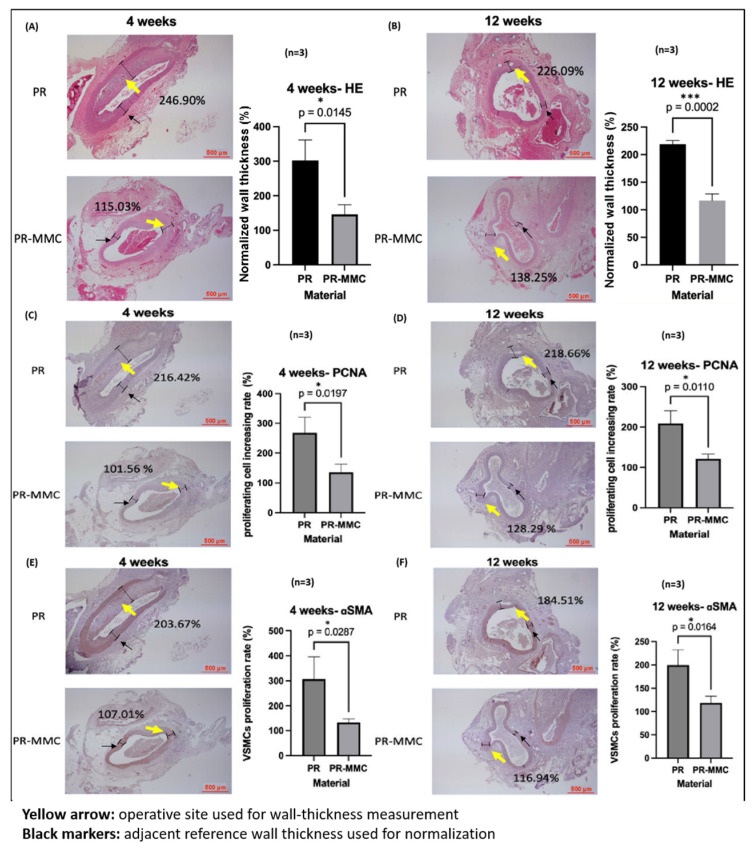
Histological evaluation of vascular wall thickening and VSMC activity at 4 and 12 weeks after vascular anastomosis using PR versus PR-MMC materials. Representative sections and quantitative analyses are shown for (**A**,**B**) H&E, (**C**,**D**) PCNA, and (**E**,**F**) αSMA staining at 4 and 12 weeks (*n* = 3 per group). Wall thickness was measured at the operative (anastomotic/treated) site (yellow arrows) from the luminal surface to the adventitial border, and values were normalized to an adjacent reference wall thickness (black markers; black arrows indicate the locations used for this reference measurement) and expressed as percentages (operative/reference × 100%). Scale bar = 500 μm. Data are presented as mean ± SD; *p* values are indicated. * *p* < 0.05, *** *p* < 0.001 (Student’s *t*-test).

Normalized wall thickness (operative/reference, %) was 243.8% (4 weeks) and 238.9% (12 weeks) in PR-group but was reduced to 132.1% and 140.2% with MMC-grafted sutures (*p* < 0.05) (Figure 5A,B).

#### 2.5.3. PCNA and α-SMA Expression

MMC immobilization reduced PCNA cell accumulation from 249.8% to 123.9% (4 weeks) and from 272.9% to 147.4% (12 weeks). α-SMA proliferation similarly declined from 275.0% to 124.0% (4 weeks) and 260.2% to 134.7% (12 weeks) (all *p* < 0.05) (Figure 5C–F). Importantly, α-SMA expression is not merely a quantitative marker of cell abundance but reflects the phenotypic transition of vascular smooth muscle cells from a contractile to a synthetic, proliferative state. The significant reduction in α-SMA-positive cells in the PR-MMC group, therefore, indicates effective inhibition of pathological phenotypic switching, a central molecular event driving intimal hyperplasia [12,13,14,15,16,41].

These results support that an interface-confined (non-eluting under the tested conditions) MMC layer effectively suppresses proliferative and synthetic VSMC phenotypes that contribute to IH.

## 3. Discussion

This study shows that covalent immobilization of MMC on polypropylene sutures creates a durable, non-releasing molecular interface that modulates VSMC behavior and attenuates IH. Plasma-activated functional groups permit stable anchoring of MMC, minimizing initial burst release and preserving mechanical integrity [33,34]. The absence of detectable MMC elution under the tested conditions supports that the therapeutic effect is likely surface-confined, arising from direct biointerface–cell interactions rather than diffusion.

### 3.1. Molecular Regulation of VSMC Fate at a Non-Releasing Interface

MMC acts by inducing DNA crosslinking, thereby activating p21, inhibiting cyclin B1–CDK1, inducing G_2_/M arrest, and promoting apoptosis [35,36,37,38,39,40,42]. Although direct quantification of p21 or cyclin B1–CDK1 signaling was not performed in this study, the observed accumulation of sub-G1 populations and suppression of proliferative markers are consistent with well-established MMC-induced molecular pathways. MMC is known to induce DNA interstrand crosslinks, activate DNA damage response signaling, upregulate p21, and subsequently inhibit cyclin B1–CDK1 complexes, thereby enforcing G_2_/M arrest and apoptosis [35,36,37,38,39,40,42]. The concordance between these canonical pathways and our cell cycle and histological findings supports a mechanism-informed interpretation of the interface-mediated effects observed herein. The dramatic shift to 66% sub-G1 VSMCs is consistent with sustained, contact-mediated apoptotic signaling at the material interface. Reduced PCNA and α-SMA expression in vivo further indicates suppression of proliferative and synthetic VSMC phenotypes, aligning with established IH mechanisms [12,13,14,15,16,41,42,43,44,45].

An important mechanistic question raised by our contact-dependent design is how a tethered MMC layer could trigger intracellular stress and DNA damage response (DDR) signaling without requiring bulk diffusion or substantial cellular uptake of free MMC. Two non-mutually exclusive mechanisms may contribute. First, interface-presented bioactive layers can directly engage the plasma membrane and induce interfacial stress signaling; for immobilized molecules, electrostatic and structural constraints at the interface can govern membrane association and downstream responses, consistent with contact dependence [28]. Second, MMC is a bioreductive quinone-containing agent that can undergo redox cycling and generate reactive oxygen species (ROS) (e.g., hydrogen peroxide) during reduction–reoxidation processes [46,47]. Notably, ROS generation has been reported not only for free MMC but also for MMC irreversibly bound to DNA, supporting the concept that localized reactive species may be produced in a proximity-confined microenvironment. Under intimate cell–material apposition, such near-surface ROS/reactive intermediates could elicit oxidative/genotoxic stress and activate DDR/checkpoint pathways (e.g., γH2AX formation and ATR (ATM and Rad3-related)/ATM (ataxia-telangiectasia mutated)-related signaling), thereby converging on p21 induction and cell cycle arrest [48]. While these hypotheses are consistent with the observed contact-dependent phenotype, future studies will quantify interface-localized ROS generation and evaluate DDR signaling (e.g., γH2AX/ATM–ATR axis) to further substantiate the proposed contact-mediated mechanism and define causal links between surface-bound MMC and downstream cell cycle arrest/apoptosis.

### 3.2. Advantages of Non-Releasing, Contact-Dependent Biointerfaces over Drug-Eluting Sutures

Prior drug-eluting sutures (paclitaxel, sirolimus, tacrolimus) provided transient suppression of IH but were limited by burst release, payload depletion, and coating instability under physiological strain [20,21,22,23,24,25]. In contrast, covalent MMC anchoring:

(i) prevents drug exhaustion;
(ii) avoids systemic exposure;
(iii) maintains uniform bioactivity throughout the healing period; and
(iv) preserves suture mechanics and surgical usability.

Collectively, these attributes define a new category of self-active biointerfaces in which therapeutic function derives from molecular surface chemistry rather than release kinetics.

A defining feature of this platform is its contact-dependent mode of action. Because MMC is not released, apoptotic signaling is restricted to cells directly in contact with the suture surface. This spatial confinement minimizes unintended exposure of surrounding healthy tissue, potentially reducing inflammatory responses and preserving endothelial regeneration in adjacent regions. Such localized molecular activity distinguishes non-releasing biointerfaces from conventional eluting systems and underscores their advantage in vascular applications where precise spatial control of cell fate is critical.

Regarding systemic safety (Table 2), modest fluctuations were observed in individual serum biomarkers (e.g., ALT in the PR–MMC group at 4 weeks). Importantly, this isolated change was not accompanied by overt clinical signs or a consistent pattern across other serum biomarkers, suggesting inter-individual variability rather than a treatment-related systemic effect. Nevertheless, larger cohorts and longer-term follow-up will be valuable for further characterizing inter-individual variability and confirming systemic safety.

Consistent with an interface-confined mechanism enabled by covalent immobilization, UV–Vis analysis (365 nm) of supernatants collected every 24 h for 14 days showed no detectable MMC signal above the blank/background under the tested static incubation conditions. This supports the conclusion that the observed bioactivity is unlikely to be attributable to the diffusion of released MMC into the bulk medium. While UV–Vis monitoring yields a non-detectable elution profile under the tested conditions, future work may employ higher-sensitivity analytical methods (e.g., high-performance liquid chromatography (HPLC) or liquid chromatography–mass spectrometry (LC–MS)) to further corroborate trace-level detection limits.

### 3.3. Translational Implications

Because polypropylene sutures are already widely used clinically, integrating this modification into existing workflows is feasible. This platform may reduce restenosis risk in coronary bypass, peripheral reconstruction, hemodialysis access creation, and microvascular surgery.

Generalizability and translation. While the present rat anastomosis model supports the bioreactivity and anti-remodeling potential of a non-releasing MMC-anchored suture interface, translation to clinical practice will require validation in larger animal models and in disease-relevant settings (e.g., diabetes or atherosclerosis), as well as evaluation under varied anastomotic configurations and longer follow-up. These studies will clarify robustness, safety margins, and manufacturability before clinical adoption.

### 3.4. Limitations and Future Directions

Sample size followed the 3Rs principle. Although results are biologically consistent, larger studies are warranted. Future work may explore co-immobilization of complementary agents such as anti-inflammatory or endothelial-promoting molecules and evaluate performance under diabetic or atherosclerotic conditions.

Endothelial healing is a key determinant of long-term patency after vascular reconstruction. An ideal anastomotic biomaterial should suppress pathological VSMC proliferation while supporting endothelial cell (EC) regeneration and function. In this study, we focused on VSMC-driven mechanisms and did not directly assess EC responses to the MMC-modified surface (e.g., viability, migration, barrier integrity, and nitric oxide-related function), which represents an important limitation. Clinically, if the suture–tissue interface contacts the luminal environment during early healing, excessive antiproliferative activity could theoretically delay re-endothelialization, a recognized safety consideration for antiproliferative vascular implants. Accordingly, future work will incorporate EC-specific in vitro assays and in vivo assessments of endothelial coverage and function to define an appropriate therapeutic window and ensure translational safety [26,27]. Because re-endothelialization is a key determinant of long-term patency after vascular reconstruction, future work will incorporate endothelial cell-specific assays to ensure that the MMC-modified interface does not delay endothelial healing.

## 4. Materials and Methods

### 4.1. Materials and Plasma Functionalization

Polypropylene sutures (PROLENE^®^ 8–0, Ethicon, Raritan, NJ, USA) were selected for their mechanical stability and clinical ubiquity [12,13,14,15,16]. Planar polypropylene membranes served as analytical analogs. Acetic acid plasma treatment was performed to introduce hydroxyl, carbonyl, and carboxyl functionalities [33,34], thereby enabling covalent MMC immobilization.

Sutures and membrane substrates were placed on the lower electrode of a custom-built low-pressure plasma reactor. The chamber was evacuated to 100 mTorr, and glacial acetic acid vapor (≥99.7%) was introduced as the working gas. Plasma discharge was applied at 50 W for 10 min, corresponding to a low-pressure, non-thermal (“cold”) plasma process. No external heating or temperature control was applied, and sample temperature was not actively monitored. Immediately after plasma treatment, substrates were immersed in an aqueous MMC solution (5 × 10^−4^ M in deionized water) and incubated for 2 h at ambient/room temperature under gentle agitation (30 rpm) to promote surface coupling. Samples were then rinsed thoroughly with deionized water to remove non-covalently bound MMC, vacuum-dried, and stored at ambient conditions until further use.

### 4.2. Physicochemical Characterization of Plasma-Activated and MMC-Grafted Surfaces

#### 4.2.1. Water Contact Angle

Surface wettability was measured by the sessile-drop method using a goniometer (Type G-1, ERMA Optical Works, Taipei, Taiwan). Five measurements were obtained per group (untreated PP and AAP-treated PP). Reduced contact angle was interpreted as increased hydrophilicity, consistent with the introduction of polar surface functionalities [49].

#### 4.2.2. FTIR

Surface chemical changes were analyzed by ATR-FTIR (Spotlight 200i; PerkinElmer, Waltham, MA, USA). Spectra were collected over 500–4000 cm^−1^ (4 cm^−1^ resolution, 16 scans). Peaks assigned to O–H (~3200–3700 cm^−1^), C=O (~1700 cm^−1^), and C–O (~1100 cm^−1^) after AAP treatment were used to indicate incorporation of oxygen-containing groups [33,34].

#### 4.2.3. SEM and EDS

Surface morphology was examined by scanning electron microscopy (SEM; JSM-7600F, JEOL Ltd., Akishima, Tokyo, Japan) at 15 kV after sputter-coating the samples with a thin platinum layer. Surface coverage of deposited features was semi-quantified using ImageJ (version 1.54g; National Institutes of Health, Bethesda, MD, USA) from representative SEM micrographs. Elemental composition was assessed using energy-dispersive X-ray spectroscopy (EDS; Bruker FQ5060, Bruker, Middlesex, NJ, USA).

Because plasma-induced oxygen functionalities on polypropylene are confined to an ultrathin near-surface layer, and light-element (C/O) signals can be dominated by the bulk polymer in EDS, SEM–EDS was not used as a primary readout to evaluate the immediate post-plasma activation state. Instead, plasma activation was validated by changes in wettability (water contact angle) and ATR–FTIR spectra.

For MMC immobilization, EDS was performed after the grafting step, and the appearance of nitrogen-related signals (absent in pristine polypropylene but present in MMC) was used as a practical marker supporting successful MMC incorporation on the treated surface.

### 4.3. Mechanical Testing

Tensile testing was conducted using a microforce testing platform (Kammrath Weiss GmbH, Dortmund, Germany) equipped with a 1 N load cell to evaluate whether plasma treatment and MMC immobilization affected suture mechanical integrity [50,51]. Polypropylene suture segments (10 cm length; needle removed) were mounted in a standardized frame (grip area: 2 mm × 1.7 mm) and stretched at a constant rate of 250 mm/min under ambient laboratory conditions until failure. Maximum load (N) and elongation at break (mm) were recorded. For each group, *n* = 5 independent specimens were tested, and representative load–displacement curves were generated.

### 4.4. Mitomycin C Elution Assessment by UV–Vis Spectroscopy

Potential MMC elution from PR-MMC sutures was evaluated using UV–Vis spectroscopy (SpectraMax iD3, Molecular Devices, San Jose, CA, USA) at 365 nm. An MMC calibration curve was established using aqueous MMC standards (0–0.50 mg/mL), yielding excellent linearity (A365 = 3.5249·C + 0.0367; R^2^ = 0.9999, where A is absorbance, and C is MMC concentration in mg/mL). For the elution test, a 45 cm length of suture was incubated in 500 μL deionized water containing lysozyme (0.5 mg/mL). Supernatants were collected every 24 h for 14 consecutive days and analyzed at 365 nm against the corresponding blank/background. MMC concentration was calculated from the calibration equation.

### 4.5. In Vitro Cytocompatibility and Cell Fate Analysis

A7r5 VSMCs [52] were co-cultured with untreated or MMC-grafted membranes using a direct-contact configuration. Briefly, PP membranes (untreated control or AAP-treated MMC-grafted) were placed at the bottom of a 12-well culture plate and secured with an O-ring to maintain stable contact. A7r5 cells were seeded at 2 × 10^4^ cells/well and incubated for 48 h under standard conditions (37 °C, 5% CO_2_). Each well contained one PP membrane secured at the bottom (contact area ≈ 3.8 cm^2^), corresponding to an initial seeding density of approximately 5.3 × 10^3^ cells/cm^2^ (2 × 10^4^ cells per well). Since the assay used a direct-contact configuration, exposure was defined by the material–cell contact area rather than by material mass. Culture medium volume was 1.0 mL per well. The water-soluble tetrazolium salt-1 (WST-1) assay was used to assess ISO 10993-5 viability. Cell cycle distribution and apoptosis (including G_2_/M and sub-G1 fractions) were quantified by PI/RNase staining (FxCycle™ PI/RNase staining solution) and flow cytometry (488 nm excitation; 620 nm emission). Cells were fixed in 75% cold ethanol prior to PI/RNase staining.

### 4.6. In Vivo Aortic Anastomosis Model and Ethical Considerations

Experimental unit: one individual rat. Male Wistar rats (10–12 weeks, 300–350 g) underwent an infrarenal aortotomy and repair with either control polypropylene sutures or MMC-grafted sutures (*n* = 3 per group per time point). Animals were obtained from an accredited vendor with standard health status documentation and had not undergone any prior experimental procedures. All animal procedures were approved by the Institutional Animal Care and Use Committee (IACUC) of Chang Gung Memorial Hospital, Taiwan (Approval No. 2020112401; approval date: 7 May 2021; valid period: 1 December 2020–30 November 2021; protocol title: “Inhibition of vessel intimal hyperplasia after anastomotic surgery by inhibition of platelet aggregation or smooth muscle cell hyperplasia”).

Animals, housing, and eligibility. Animals were housed at the Keelung Chang Gung Memorial Hospital Animal Center, an Association for Assessment and Accreditation of Laboratory Animal Care International (AAALAC) -accredited facility, under standard controlled conditions with ad libitum access to food and water. Animals were pair-housed before surgery and singly housed postoperatively to facilitate recovery and to prevent wound interference, in accordance with institutional animal care guidelines. No a priori inclusion/exclusion criteria were set, and no animals or data points were excluded from analysis.

Anesthesia, analgesia, monitoring, and humane endpoints. Procedures were performed under inhalational anesthesia with isoflurane delivered via a precision vaporizer (3% for induction and 2% for maintenance) in oxygen (1 L/min for induction and 0.5 L/min for maintenance; Piramal Critical Care, Bethlehem, PA, USA), with local infiltration of 0.2 mL of 1% lidocaine (AstraZeneca Pty Ltd., Macquarie Park, NSW, Australia). Perioperative supportive care and analgesia were provided in accordance with the IACUC-approved protocol and veterinary guidance. Postoperative analgesia was provided by ibuprofen oral suspension (20 mg/mL; 3.0 mL added to 300 mL drinking water, final concentration 0.2 mg/mL), supplied ad libitum for 3 days postoperatively, and replaced in accordance with institutional animal care guidelines. Animals were monitored at least daily for general condition, activity, wound integrity, and signs of pain or distress. Humane endpoints were applied to prevent unnecessary suffering, including persistent severe pain/distress despite supportive care, inability to access food or water, marked or sustained weight loss, severe infection or wound dehiscence, uncontrolled bleeding, respiratory distress, or moribund condition, as determined by veterinary staff.

Surgical procedure and tissue harvest. After midline laparotomy, the infrarenal abdominal aorta was exposed and clamped proximally and distally. An ~8 mm longitudinal aortotomy was created and repaired using three simple interrupted stitches with either unmodified polypropylene sutures (PR) or MMC-grafted sutures (PR–MMC). The total aortic clamp time was kept to less than 3 min. Hemostasis was achieved by brief gauze compression, and the abdominal wall was closed in layers. Postoperatively, animals were rewarmed under a heat lamp until fully recovered from anesthesia. Systemic heparinization was not performed. At postoperative weeks 4 and 12, animals were anesthetized and euthanized by exsanguination, followed by tissue harvest. Anastomotic segments were collected for histological and immunohistochemical analyses (PCNA and α-SMA) [41,43,44]. Systemic safety was evaluated using hematologic and biochemical panels.

Outcome measures and blinding. The in vivo assessment was designed as a proof-of-concept evaluation of bioreactivity and vascular remodeling. The primary outcome was arterial wall thickness at the anastomosis site at 4 and 12 weeks. Secondary outcomes included PCNA and α-SMA immunohistochemical quantification and systemic safety parameters (hematology and serum biochemistry). Outcome assessment (histology and immunohistochemical quantification of PCNA and α-SMA) was performed by an assessor blinded to group allocation. Investigators were not blinded during surgery, perioperative care, or group allocation.

Normalization of histological readouts. For all histology-derived percentage metrics reported in this study (e.g., wall thickness, PCNA-associated proliferative index, and αSMA-associated VSMC index), values were calculated as the ratio of the operative-site measurement to an adjacent reference measurement within the same section and expressed as percentages (operative/reference × 100), with the reference defined as 100%.

Study design and sample size rationale. Randomization was not performed. The in vivo experiment was designed as a proof-of-concept confirmation of in vivo bioreactivity following extensive in vitro and physicochemical characterization; accordingly, the sample size was minimized in line with the 3Rs principles (Replacement, Reduction, and Refinement), using *n* = 3 per group per time point to confirm the direction and consistency of tissue responses and to screen for overt in vivo safety concerns or adverse tissue reactions.

### 4.7. Statistical Analysis

Statistical analyses were performed using GraphPad Prism 9 (GraphPad Software, San Diego, CA, USA). Quantitative data are presented as mean ± standard deviation (SD), for comparisons between two groups, unpaired two-tailed Student’s *t*-tests were used. Group comparisons (PR vs. PR–MMC) were performed separately at each time point (4 and 12 weeks). For comparisons involving more than two groups, one-way analysis of variance (ANOVA) followed by Tukey’s post hoc test was applied; specifically, the multi-group comparison of wettability changes following plasma exposure (Figure 1D) was analyzed using one-way ANOVA with Tukey’s correction.

Given the relatively small sample size in the in vivo study (*n* = 3 per group per time point), statistical analysis was intended to support an exploratory, proof-of-concept evaluation; therefore, results were interpreted in conjunction with consistency across related biological readouts (e.g., arterial wall thickness and immunohistochemical quantification of PCNA and α-SMA). No data points were excluded from analysis. A two-sided *p*-value < 0.05 was considered statistically significant.

## 5. Conclusions

Covalent MMC immobilization on polypropylene sutures creates a non-releasing molecular biointerface that exerts sustained, contact-mediated regulation of VSMC fate. This interface suppresses G_2_/M progression, induces apoptosis, and significantly reduces IH in vivo without overt evidence of systemic toxicity. By transforming a passive surgical material into an active therapeutic device, this strategy offers a clinically practical, mechanism-informed approach to preventing pathological vascular remodeling.

## Figures and Tables

**Table 1:** Elemental composition of polypropylene membranes and sutures before and after MMC immobilization, determined by energy-dispersive spectroscopy (EDS). Nitrogen signals detected in MMC-grafted samples confirm successful molecular anchoring.

Atomic %	C	O	N
PP	99.71	0.29	0.00
PP-MMC	78.60	8.70	12.0
Atomic %	C	O	N
PR	99.84	0.16	0.00
PR-MMC	74.68	20.63	4.69

**Table 2:** Hematological and biochemical parameters of rats before surgery and at 4 and 12 weeks following vascular repair with unmodified or MMC-grafted sutures. All values were within or close to normal physiological ranges, without overt evidence of hepatic or renal toxicity. Abbreviations: WBC, white blood cell; RBC, red blood cell; ALT, alanine aminotransferase; AST, aspartate aminotransferase.

Blood Test (Normal Range)	Pre-Op	PR-4 Week	PR-MMC-4 Week	PR-12 Week	PR-MMC-12 Week
WBC (5.5~10 × 10^3^/µL)	7.6 ± 2.4	9.4 ± 2.1	8.1 ± 3.2	7.6 ± 0.3	8.4 ± 1.1
RBC (6.9~8.3 × 10^6^/µL)	7.4 ± 1.3	6.9 ± 0.7	7.3 ± 0.4	8.3 ± 0.6	7.5 ± 0.5
Hemoglobin (13.7~17.6 g/dL)	14.0 ± 2.6	13.8 ± 1.4	13.7 ± 0.6	15.9 ± 1.0	13.9 ± 0.9
Platelets (638~1177 × 10^3^/µL)	991 ± 377.6	976 ± 357.0	1045 ± 144.0	1246.7 ± 18.0	908 ± 109.3
ALT (25~89 U/L)	40.5 ± 6.3	37.7 ± 5.1	61.1 ± 25.1	34.7 ± 5.7	48.0 ± 11.4
AST (65~203 U/L)	116.8 ± 37.1	66.1 ± 16.1	82.4 ± 45.3	72.6 ± 19.2	103.3 ± 11.5
Creatinine (0.3~1.0 mg/dL)	0.4 ± 0.1	0.4 ± 0.1	0.6 ± 0.1	0.4 ± 0.1	0.5 ± 0.0
Albumin (3.4~4.8 g/dL)	4.1 ± 1.5	3.62 ± 0.1	3.99 ± 0.27	3.84 ± 0.15	3.67 ± 0.43

## Data Availability

The data presented in this study are available on reasonable request from the corresponding author.

## Abbreviations

The following abbreviations are used in this manuscript:

AAP	Acetic-acid plasma
AAALAC	Association for Assessment and Accreditation of Laboratory Animal Care International
ALT	Alanine aminotransferase
ANOVA	analysis of variance
AST	Aspartate aminotransferase
ATM	ataxia-telangiectasia mutated
ATR	ATM and Rad3-related
ATR-FTIR	attenuated total reflection Fourier-transform infrared spectroscopy
DNA	Deoxyribonucleic acid
DDR	DNA damage response
EDS	Energy-dispersive spectroscopy
FTIR	Fourier-transform infrared spectroscopy
G_2_/M	G2/M phase (cell cycle checkpoint)
H&E	hematoxylin and eosin
HPLC	high-performance liquid chromatography
IACUC	Institutional Animal Care and Use Committee
IH	Intimal hyperplasia
ISO	International Organization for Standardization
LC–MS	liquid chromatography–mass spectrometry
MMC	Mitomycin C
PCNA	Proliferating cell nuclear antigen
PI	propidium iodide
PP	Polypropylene (membrane)
PP–MMC	Mitomycin C–grafted polypropylene (membrane)
PR	Polypropylene suture (PROLENE^®^)
PR–MMC	Mitomycin C–grafted polypropylene suture
RBC	Red blood cell count
ROS	reactive oxygen species
SD	Standard deviation
SEM	Scanning electron microscopy
UV–Vis	ultraviolet–visible
VSMC(s)	Vascular smooth muscle cell(s)
WBC	White blood cell count
WST-1	Water-soluble tetrazolium salt-1 assay
α-SMA	Alpha-smooth muscle actin
